# Loss of CADM1 expression is associated with poor prognosis and brain metastasis in breast cancer patients

**DOI:** 10.18632/oncotarget.1832

**Published:** 2014-03-16

**Authors:** Harriet Wikman, Laura Westphal, Felicitas Schmid, Sirkku Pollari, Jolanthe Kropidlowski, Bettina Sielaff-Frimpong, Markus Glatzel, Jakob Matschke, Manfred Westphal, Kristiina Iljin, Heini Huhtala, Luigi Terracciano, Anne Kallioniemi, Guido Sauter, Volkmar Müller, Isabell Witzel, Katrin Lamszus, Dirk Kemming, Klaus Pantel

**Affiliations:** ^1^ Institute of Tumor Biology, University Medical Center Hamburg-Eppendorf, Hamburg, Germany; ^2^ Max-Delbrück Center for Molecular Medicine, Berlin, Germany; ^3^ Medical Biotechnology, VTT Technical Research Centre of Finland and Turku Centre for Biotechnology, University of Turku, Turku, Finland; ^4^ Institute of Neuropathology, University Medical Center Hamburg-Eppendorf, Hamburg-Eppendorf, Hamburg, Germany; ^5^ Department of Neurological Surgery, University Medical Center Hamburg-Eppendorf, Hamburg-Eppendorf, Hamburg, Germany; ^6^ School of Health Sciences, University of Tampere, Tampere, Finland; ^7^ Department of Pathology, Basel University Clinics, Basel, Switzerland; ^8^ Institute of Biomedical Technology, University of Tampere and BioMediTech, Tampere, Fimlab Laboratories, Tampere, Finland; ^9^ Institute of Pathology, University Medical Center Hamburg-Eppendorf, Hamburg, Germany; ^10^ Department of Gynecology, University Medical Center Hamburg-Eppendorf, Hamburg, Germany; ^11^ European Laboratory Association, Ibbenbueren, Germany

**Keywords:** breast cancer, brain metastases, CADM1, methylation

## Abstract

Breast cancer brain metastases (BCBM) are detected with increasing incidence. In order to detect potential genes involved in BCBM, we first screened for genes down-regulated by methylation in cell lines with site-specific metastatic ability. The expression of five genes, *CADM1*, *SPARC*, *RECK*, *TNFAIP3* and *CXCL14*, which were also found down-regulated in gene expression profiling analyses of BCBM tissue samples, was verified by qRT-PCR in a larger patient cohort. *CADM1* was chosen for further down-stream analyses. A higher incidence of *CADM1* methylation, correlating with lower expression levels, was found in BCBM as compared to primary BC. Loss of CADM1 protein expression was detected most commonly among BCBM samples as well as among primary tumors with subsequent brain relapse. The prognostic role of CADM1 expression was finally verified in four large independent breast cancer cohorts (n=2136). Loss of CADM1 protein expression was associated with disease stage, lymph node status, and tumor size in primary BC. Furthermore, all analyses revealed a significant association between loss of CADM1 and shorter survival. In multivariate analyses, survival was significantly shorter among patients with CADM1-negative tumors. Loss of *CADM1* expression is an independent prognostic factor especially associated with the development of brain metastases in breast cancer patients.

## INTRODUCTION

Breast cancer (BC) is the most common non-skin malignancy in women affecting about 1.2 million women in the world each year. The spread of malignant cells from the primary tumor to distant organs such as the brain is the main cause of BC related deaths. Breast cancer is the second most common cause for the development of central nervous system (CNS) metastases, which are, irrespectively of the primary tumor origin, more commonly diagnosed than primary brain tumors [1, 2]. CNS-metastases are diagnosed in 15-20% of patients with metastatic BC and are usually occurring with a median time period of 31 months after the diagnosis of breast cancer [2]. Due to improved systemic treatment options for breast cancer, patients benefit from prolonged survival rates. However, consecutively to the longer survival and an intensified use of sensitive detection methods such as contrast-enhanced magnetic resonance imaging of the brain (cMRI), the incidence rates of brain metastases are rising.

Brain metastases are often associated with the aggressive triple negative breast cancer (TNBC), being hormone receptor (HR) and HER2 negative, and also with HER2 positive primary breast cancers [3, 4]. Patients suffering from breast cancer brain metastases (BCBM) have an extremely poor prognosis with a median survival of only 7.8 months [3]. This brings forth to investigate new prognostic markers, which may be helpful for the identification of patients with an increased risk for subsequent CNS-infiltration and to develop new therapeutic approaches.

The loss of tumor suppressor gene (TSG) expression is known to constitute a crucial step in cancer formation. For the dissemination and outgrowth of metastases, another set of metastasis suppressor and activator genes is needed. Metastasis suppressor genes (MSGs) usually do not influence tumor growth at the primary site, but control the tumor cells' capacity to escape from the primary tumor and form overt metastases at distant sites [5]. MSGs contribute to dormancy control and/or outgrowth at secondary sites and thus regulate the final step of metastasis, the metastatic colonization. Interestingly, in contrast to TSGs, MSGs seem to be more often cancer type specific. Furthermore, MSGs are rarely mutated, and epigenetic events, such as methylation, are thus likely the main cause for their loss of function [6]. Since epigenetic events are reversible, dormancy control by MSGs is potentially a new form of targeted gene therapy (reviewed in [6, 7]).

The aim of this study was to identify novel genes involved in BCBM formation. We first screened for genes down-regulated by methylation in breast cancer cell lines (parental MDA-MB-231 cell line and the brain- and bone-specific sub cell lines) known for site-specific metastasis [8]. Gene expression profiling and quantitative real-time RT-PCR (qRT-PCR) validation of the candidate genes were thereafter conducted on tumor tissues from primary BC and BCBM in order to identify the most clinically relevant genes. The methylation pattern of *CADM1* was furthermore characterized, and CADM1 protein expression was validated in two large independent primary tumor cohorts as well as in BCBM samples and correlated with clinico-pathological parameters.

## RESULTS

### Methylation array screening of brain metastases related genes

A subclone of MDA-MB-231 with a high metastatic potential to the brain, MDA-MB-231 BR, was compared to the parental MDA-MB-231 and to a bone-seeking variant MDA-MB-231 SA in order to identify genes, which might be specifically involved in brain metastasis formation. The cell lines were treated with 5-Aza-2'-deoxycytidine, a demethylating agent, in order to find genes potentially down-regulated by methylation. Microarray analysis was performed on pooled triplicate experiments and the non-tumorigenic epithelial cell line MCF 10A was used to control for stress response after the treatment with 5-Aza-2'-deoxycytidine.

**Figure 1:**
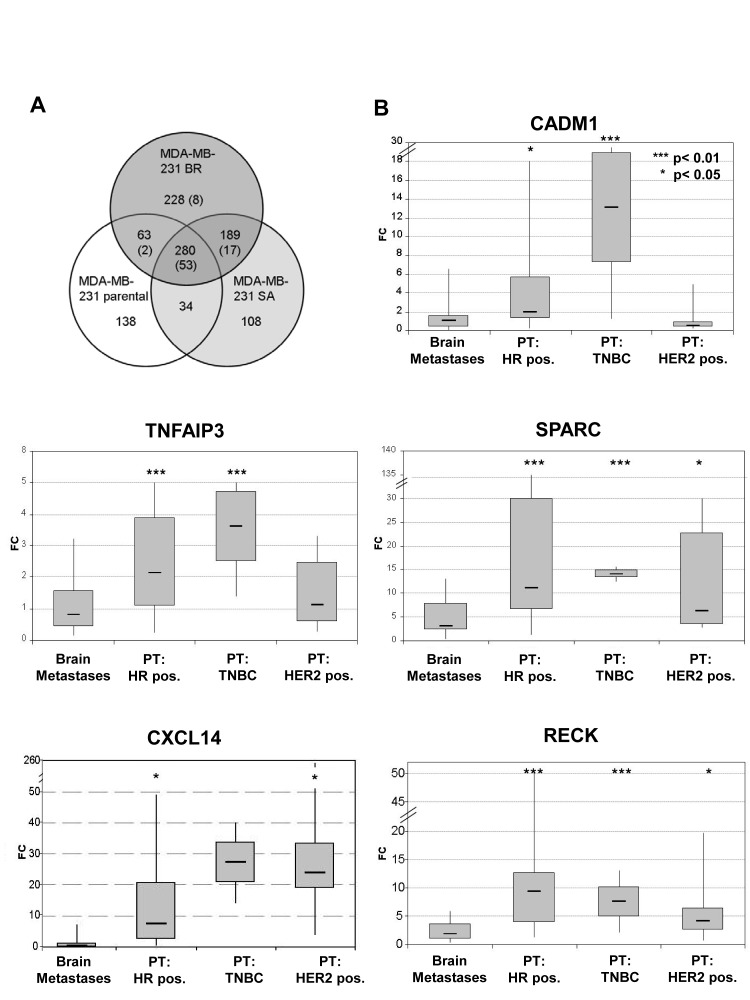
A) Gene expression changes in response to 5-Aza-2'-deoxycytidine treatment in parental MDA-MB-231 breast cancer cell line and MDA-MB-231 BR and MDA-MB-231 SA variants The number before brackets defines the genes up-regulated in each cell line after 5-Aza-2'-deoxycytidine treatment. The number in brackets defines the number of genes also down-regulated in the brain metastases as compared to non-relapsed primary tumors. Figure 1B) Expression of *CADM1*, *RECK*, *CXCL14*, *SPARC* and *TNFAIP3* in BCBM samples relative to primary breast tumors (PT). HR pos.: estrogen and progesterone positive receptor, TNBC: triple negative breast cancer. P- values were determined by the log rank test.

The gene expression profiling after 5-Aza-2'-deoxycytidine treatment revealed 914 different transcripts, which were significantly up-regulated in one of the MDA-MB-231 cell lines but not altered in MCF 10A (Figure 1A). The largest number of up-regulated genes (691 transcripts) and cell line-specific up-regulated genes (20%) was found in the MDA-MB-231 BR cell line. Most of the genes were, however, up-regulated in all of these cell lines (30%, 279/914). In general, the more aggressive subclones BR and SA were more similar to each other than to the parental cell line, indicating a differentiation into generally more aggressive forms.

### Gene expression screening for methylation-related genes in primary and metastatic breast tumors

Gene expression profiling of primary non-metastasized breast tumors (n=32) and brain metastases (n=9) was performed to find the most relevant genes among the 690 potentially brain metastases determining genes detected in methylation array screening. 110 transcripts (16%) of those up-regulated in the MDA-MB-231 BR subclone in response to 5-Aza2'-deoxycytidine treatment were found significantly lower expressed among the BCBM samples as compared to the samples from non-relapsed primary BC patients (Supplementary Table 2).

Twenty-four (22%) of these genes were found exclusively in the BR subclone, implicating a brain specific down-regulation of these genes. 28 genes were in common between the highly metastatic BR and SA variants, indicating that these genes may mediate a more aggressive behavior in general.

### Validation of potential metastasis-suppressing genes in primary BC and BCBM tissue samples

The expression of five genes, which we found down-regulated in the breast cancer data set and in the cell line analyses, was further investigated by qRT-PCR in 39 primary BC samples without brain metastases and 20 BCBM tissue samples.

*SPARC, RECK, TNFAIP3* and *CXCL14* were down-regulated (p < 0.05) in BCBM as compared to primary BC samples irrespectively of the cancer subtype, while for *CADM1*, this correlation was found in HR positive and TNBC samples (Figure 1B and Supplementary Figure 1). Within the primary BC samples, 84% of the HER2 negative patients showed an elevated *CADM1* mRNA expression (top 75% percentile), whereas only 29% of the HER2 positive patients had a high *CADM1* expression (p = 0.003) (Supplementary Figure 1). Among the primary tumors, high RECK expression was also associated with HR positive status, whereas the highest *SPARC* expression was statistically significantly linked to triple negative (TNBC) samples. *CXCL14* expression was not associated with a subtype, but its low expression was associated with both positive lymph node status (p = 0.011) and high grade (p = 0.029) (Supplementary Table 3).

### Frequency of *CADM1* methylation in primary breast cancer and BCBM tissue samples

**Figure 2:**
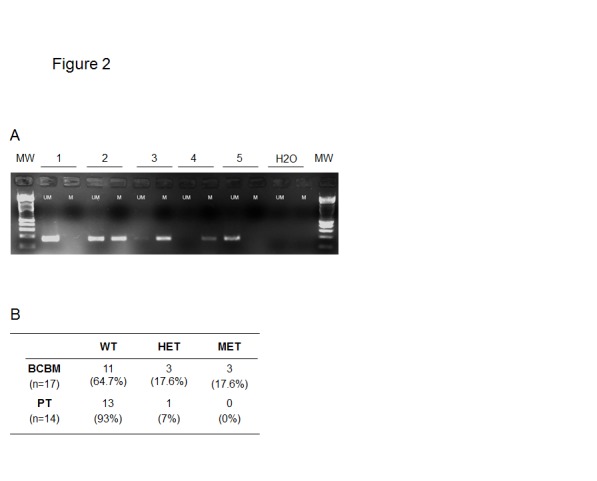
Promoter CpG methylation of *CADM1* A) Gel electrophoresis picture of *CADM1* in 5 representative cases, MW= molecular weight marker, UM= unmethylated PCR, M= methylated PCR. B) Tables of MSP results for *CADM1* of primary tumors and breast cancer brain metastases. WT= wild type, HET= heterozygous methylation, MET= homozygous methylation.

The methylation status of *CADM1* was determined by MSP in 17 BCBM and 14 primary BC samples. *CADM1* was homozygously methylated in 17.5% (3/17), heterozygously methylated in 17.5% (3/17) and not methylated in 65% (11/17) of the BCBM samples. In contrast, primary BC samples showed no homozygous *CADM1* methylation, only 7% (1/14) heterozygous and 93% (13/14) WT *CADM1* status (Figure 2). Due to the low frequency of methylation in the primary BC samples, no association between clinico-pathological factors and the methylation status of *CADM1* could be found.

### CADM1 protein expression in primary and metastatic breast cancer

**Figure 3:**
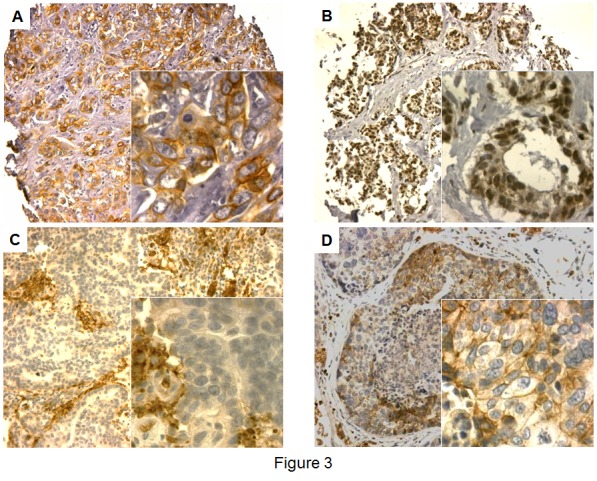
CADM1 immunostaining in primary breast cancer and BCBM samples A) Primary BC sample with homogenous positive CADM1 membrane and negative nuclear staining, B) Primary BC sample with negative CADM1 membrane and weak nuclear staining, C) BCBM sample with negative CADM1 membrane and nuclear staining with positively stained erythrocytes, D) BCBM sample with weak heterogeneous CADM1 membrane and negative nuclear staining.

CADM1 protein expression could be assessed in a large number of primary breast tumors (TMA I, n= 1331) and 27 BCBM samples (Table 1, and Figure 3). Normal breast duct showed an intensive membranous staining for CADM1, whereas the tumor stroma was CADM1 negative. The CADM1 expression in the plasma membrane was lost in 42% of the primary BC and in 68% of the BCBM samples. Strong expression was recorded in 22% of the primary BC and in 11% of the BCBM cases. The CADM1 protein expression differed between primary tumor and BCBM with more negative staining among the BCBM samples (p = 0.011).

**Table 1:** CADM1 membrane protein expression in correlation to clinical parameters in primary BC (TMA I) and BCBM

		CADM1 negative		CADM1 weak		CADM1 strong		p- value
		n	%	n	%	n	%	
Brain metastases								
All		19	67.9	6	21.4	3	10.7	
Primary tumors								
All		557	42.1	488	36.9	286	21.6	
Histology								n.s.
	Ductal	408	42.1	361	37.2	201	20.7	
	Lobular	71	46.4	51	33.3	31	20.3	
	others	78	37.7	76	35.2	54	27.1	
Tumor stage								<0.001
	pT1	118	27.6	185	43.0	125	29.0	
	pT2	290	44.8	224	34.6	133	20.6	
	pT3	40	52.6	25	32.9	11	14.5	
	pT4	106	60.6	52	29.7	17	9.7	
	n.a.	3		2		0		
Lymph node status								0.003
	pN0	193	35.8	219	40.6	127	23.6	
	pN1	207	44.6	159	34.3	98	21.1	
	pN2	43	53.8	28	35.0	9	11.3	
	n.a.	114		82		52		
Grade								n.s.
	1	130	42.6	113	37.0	62	20.3	
	2	201	40.4	188	37.8	108	21.7	
	3	203	45.8	151	34.1	89	20.1	
	n.a.	23		36		27		
Tumor size								<0.001
	< 2.0 cm	120	27.9	185	43.0	125	29.1	
	> 2.0 cm	421	48.4	290	33.3	159	18.3	
	n.a.	16	2.9	13	2.7	2	0.7	
Hormone receptor								n.s.
	negative	126	44.3	87	32.8	65	22.9	
	positive	410	41.0	379	37.9	210	21.0	
	n.a.	21		22		11		
HER2								n.s.
	negative	438	40.4	403	37.2	242	22.3	
	positive	93	45.6	73	35.8	38	18.6	
	n.a.	26		12		6		
Subtype								0.036
	ER/PR pos.	350	40.4	332	38.3	185	21.3	
	TNBC	81	42.4	55	28.8	55	28.8	
	HER2 pos.	93	45.6	73	35.8	38	18.6	
	n.a.	33		28		8		
Age								0.028
	< 50 years	85	37.1	85	37.1	59	25.8	
	> 50 years	379	44.8	310	36.6	157	18.6	
	n.a.	93		93		70		
Course of disease								<0.001
	dead	238	51.7	145	31.7	76	16.6	
	alive	319	36.6	343	39.3	210	24.0	

n.a. = not available; n.s. = not significant

Surprisingly, a nuclear staining of CADM1 was seen in a small subset of patients. Nuclear staining has been previously reported to occur in cervical epithelium with the localization of staining being dependent on epithelial origin [9]. 86 (6.5%) of the primary BC samples on the prognostic TMA (TMA I) had a clear CADM1 staining in the nucleus. The nuclear staining highly correlated with the membranous staining (p < 0.001). In 70 of the 86 patients with nuclear staining, also the membrane was stained for CADM1 (81% concordance).

In addition, 43 matched pairs of primary tumors and lymph node metastasis samples (TMA III) were analyzed for CADM1 protein expression. 70% (30/43) of the matched pairs showed a concordant CADM1 expression with 51% (22/43) of the primary tumor and lymph node samples being CADM1 negative and 19% (8/43) CADM1 positive in both types of tissues. Only 7% (3/43) of the samples had a higher CADM1 expression in the lymph nodes as compared to the matched primary tumors, while down-regulation of CADM1 expression in the lymph node metastases was observed in 23% (10/43) of the matched pairs, indicating a loss of CADM1 protein expression during the metastatic cascade.

### Comparison of gene expression, methylation pattern and protein expression status of CADM1 in primary BC and BCBM

In order to find out how much of the silencing of expression is governed by promoter methylation, we compared the results from the qRT-PCR, IHC and methylation analyses. In 22 samples results from the gene expression, methylation and protein expression status for CADM1 were available (Table 2).

**Table 2:** Combined results from the qRT-PCR, MSP, and IHC analyses for CADM1

CADM1				
Pat. No.	Sample	qPCR	methylation	IHC-result
BrM-9	BCBM	INT	MET	NEG
BrM-10	BCBM	LOW	MET	NEG
BrM-8	BCBM	INT	HET	NEG
BrM-7	BCBM	LOW	HET	NEG
BrM-5	BCBM	LOW	WT	NEG
BrM-25	BCBM	LOW	WT	NEG
BrM-19	BCBM	LOW	WT	NEG
BrM-1	BCBM	INT	WT	NEG
BrM-16	BCBM	INT	WT	NEG
BrM-18	BCBM	INT	WT	NEG
BrM-22	BCBM	INT	WT	NEG
BrM-2	BCBM	INT	WT	NEG
PT-95	PT	INT	WT	NEG
PT-103	PT	INT	WT	NEG
BrM-13	BCBM	n.d.	WT	NEG
BrM-21	BCBM	INT	HET	WEAK
PT-88	PT	INT	HET	WEAK
BrM-15	BCBM	INT	n.d.	WEAK
BrM-14	BCBM	HIGH	n.d.	WEAK
BrM-24	BCBM	LOW	WT	WEAK
PT-101	PT	INT	WT	STRONG
BrM-11	BCBM	HIGH	WT	STRONG

BCBM: breast cancer brain metastase; PT: primary tumor; HIGH: CT expression value in upper quartile in all patients analyzed; LOW: CT expression value in bottom quartile in all patients analyzed n.d.: not defined; WT: wild type; HET: heterozygous methylation; MET: homozygous methylation; NEG: negative protein staining

Homozygous methylation was associated with a negative *CADM1* staining and low or intermediate mRNA expression. Heterozygote methylation pattern showed a negative IHC result in 50% of the cases and for the rest a weak positive CADM1 protein staining was observed. Negative CADM1 protein expression was always associated with either low/ negative or intermediate mRNA expression.

### Clinical significance of CADM1 protein expression

**Figure 4:**
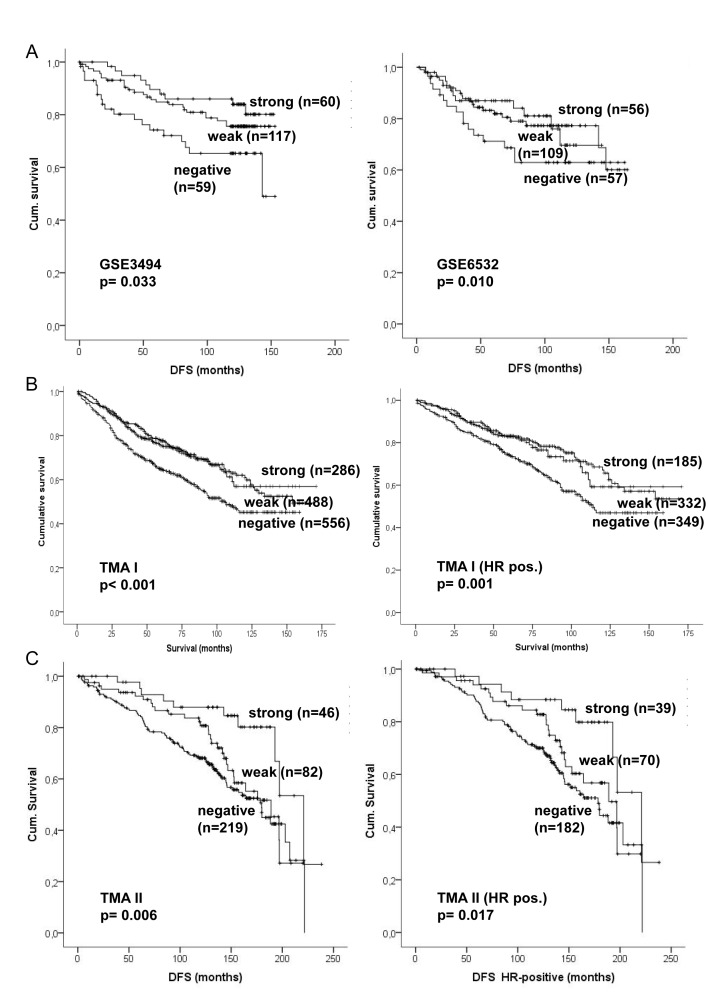
Kaplan–Meier analysis of CADM1 mRNA and protein expression in primary breast cancer A) Survival analyses in two publicly available expression data sets. B) Survival analyses for the prognostic TMA (TMA I) of the whole study population and among HR-positive patients. C) Survival analyses in the second TMA (TMA II) for the whole study population and among HR-positive patients. Survival differences were analyzed by the log rank test.

The clinical significance of *CADM1* expression was assessed in two publicly available mRNA expression data sets (GSE3494 and GSE6532) and on two prognostic TMAs of primary BC samples (Figure 4). In both expression array data sets a significant association between low *CADM1* expression and bad prognosis was found (p = 0.033 and p = 0.001).

The two prognostic TMAs (TMA I and TMA II) of primary BC gave highly consistent results. Clinico-pathologic examination of both independent patient cohorts consisting of 1718 primary breast tumor samples revealed a significant association between negative CADM1 status and advanced tumor stage, positive lymph node status and larger tumor size (all p < 0.05; Table 1 and Supplementary Table 4). In the TMA I a significant association between CADM1 and age, subtype and course of disease was also detected. The frequency distribution of CADM1 was comparable on the TMA II but significance was not reached due to smaller sample numbers. Grade was associated with CADM1 loss on the second TMA, whereas no difference between the different histological subtypes could be found on either TMA.

The site of relapse was recorded for 353 primary tumor patients (TMA II). Among patients without relapse a loss of CADM1 was seen in 58.6%, whereas the highest frequency of CADM1 loss was seen among patients suffering from brain relapse with 81.1%. Also other types of relapse had a higher frequency of CADM1 loss compared to the non-relapsed (bone 73.8% (p = 0.023), liver 69.7% and lung 65.5%; Supplementary table 4).

Consistent with the membranous staining, a statistically significant association between loss of CADM1 in the nucleus and more aggressive or advanced tumor stage and size, as well as for the hormone receptor status and the course of disease (p < 0.05) was found (only assessed on TMA I, Supplementary Table 5). In addition, the majority of cases with nuclear CADM1 staining was found among lobular tumors (11% vs. 6% in ductal, p = 0.042).

A highly significant association between worse patient outcome (overall survival and disease free survival) and negative CADM1 protein expression could be found in both data sets (p < 0.001 and p = 0.006) (Figure 4). Loss of CADM1 was significantly associated with worse patient outcome among ductal carcinomas (p = 0.001 and p = 0.001), whereas a significant association was found for lobular carcinomas only in TMA II (p = 0.026; TMA I p = 0.068) (data not shown). Furthermore, loss of CADM1 expression was significantly associated with shorter survival in HR positive (both data sets p = 0.001 and p = 0.022, Figure 4), but did not have a prognostic relevance in HER2 positive patients, indicating a tumor suppressing effect of CADM1 in HER2 negative patients only (data not shown). Multivariate analysis showed that loss of CADM1 expression was a significant independent negative prognostic factor (TMA I p = 0.045 and TMA II p = 0.01). For the first larger study cohort (TMA I) the five-year survival was 64.8% (median 105 months) for patients with no CADM1 protein expression and 75.9% (154 months) and 77.7% for patients with weak and strong CADM1 expression respectively. For TMA II the five-year survival was 84.7% (median 179.7 months) for patients with no CADM1 protein expression and 92.3% (189 months) and 95.2% (221 months) for patients with weak and strong CADM1 expression, respectively.

## DISCUSSION

The incidence of brain metastases in breast cancer patients is increasing, with no standard diagnostic management of these patients applied in clinical routine. In view of the increasing success of targeted therapies in treating BC patients with distant metastases, improved insights into the phenotype of brain metastases could have important therapeutic implications. In the present study, we first screened for genes silenced by methylation in a breast cancer cell line with a high potential to form brain metastases. Five of the identified genes, also down-regulated in clinical samples, *CADM1, RECK, SPARC, CXCL14* and *TNFAIP3*, were chosen for further expression analyses. These genes have previously not been associated with brain metastasis formation. However, they have been implicated as cancer-relevant genes in epithelial tumors [10-14]. Furthermore, the methylation status of *CADM1* in primary BC and BCBM was investigated and CADM1 protein expression was examined in correlation with clinico-pathological parameters in BC and compared to BCBM tissue samples. We identified CADM1 as an important prognostic factor, whose loss was not only associated with more aggressive primary BC with worse outcome, but also with an increased risk of brain metastasis formation.

CADM1/TSLC1 (cell adhesion molecule 1) is a membrane-spanning glycoprotein belonging to the superfamily of immunoglobulin cell adhesion molecules. It was first recognized as a tumor suppressor in non-small cell lung cancer (NSCLC) [10]. It has been suggested that the disruption of cell adhesion through the loss of CADM1 is a mechanism leading to cancer cell invasion and metastasis [15, 16]. CADM1 is known to be involved in inhibition of cell proliferation and induction of apoptosis with a reported loss of expression in a variety of cancers of epithelial cell origin such as breast, prostate, pancreatic, hepatocellular and colorectal cancer, but also in neuroblastoma (reviewed in [16]). Recently, *CADM1* was identified by Faraji et al. (2012) to be a metastasis susceptibility gene, *i.e.* an inherited factor that suppresses metastasis by sensitizing tumor cells to immunosurveillance by CD8+ T-cells [17].

We found a down-regulation of *CADM1* both on mRNA and protein level in BCBM tissue samples as compared to primary BC. It was previously reported that CADM1 protein is detected on the cell membrane in normal epithelial cells of the breast and that negative CADM1 staining, detected in more than half of the primary BCs, is associated with advanced disease stages [18, 19]. Here, we found that in primary BC, loss of CADM1 was most commonly seen among TNBC and HER2 positive patients, two subtypes more closely associated with brain metastases. Furthermore, loss of CADM1 protein expression was associated with risk factors such as high tumor stage, positive lymph node status and large tumor size (all p < 0.05) in two large independent sample cohorts consisting of more than 1 700 tumor samples, indicating a prognostic role for CADM1 in preventing breast cancer progression. Loss of CADM1 expression was found more commonly among primary tumor patients with lung, liver, bone or brain relapse compared to patients with no relapse. Importantly, loss of CADM1 expression was most commonly seen among patients with brain relapse indicating a special but not exclusive role of CADM1 in brain metastasis formation. Similarly, matched pairs of primary BC and lymph node (LN) metastasis samples were additionally analyzed for CADM1 protein expression, showing more frequently a down-regulation of CADM1 expression in the LN metastases. The observed down-regulation of CADM1 not only in BCBM but also in LN metastases as compared to the primary tumor supports the role of CADM1 as a metastasis susceptibility gene in terms of reducing the metastatic capability (25). This hypothesis is further supported by our results showing an association between CADM1 positive staining in primary tumors and longer overall survival. Multivariate analysis showed that CADM1 expression is an independent prognostic marker, strengthening the hypothesis that CADM1 may play an important role in preventing the metastatic progression.

In line with the results from our expression studies, the *CADM1* promoter region was found methylated in 35% of the BCBM samples, whereas in primary BC samples with good prognosis, *CADM1* promoter was only methylated in 7% of the samples. However, other mechanisms resulting in a loss of gene function, such as deletion, need to be further investigated. Clearly, the predictive value for CADM1 in brain metastases formation needs to be tested on large matched samples sets in future studies.

In addition to CADM1, we also analyzed the expression of four other genes (*RECK, SPARC, TNFAIP3* and *CXCL14*) in primary BC and BCBM. All four genes were found down-regulated in BCBM patients as compared to primary BC. Further studies will be needed in order to assess their biological and clinical role in brain metastases formation. RECK protein is capable of inhibiting matrix metalloproteinases and plays an important role in embryogenesis and vasculogenesis [11]. RECK is often down-regulated in various primary epithelial cancers and gliomas, and the down-regulation correlates with poor prognosis (reviewed in [11, 20]). Recently, Hill et al (2011) showed that RECK down-regulation by methylation in BC is associated with relapse and poor survival [21]. Furthermore, Hsu et al. showed that HER2 can repress RECK expression in order to promote cell invasion, which is consistent with our results showing the lowest expression of RECK among HER2 positive patients [22].

SPARC/ osteonectin (secreted protein acidic and rich in cysteine) is a matricellular calcium-binding glycoprotein involved in cell adhesion, cell-matrix interactions during tissue remodelling, cell proliferation, migration, angiogenesis and apoptosis [12, 23]. Recently, Nagai et al. [24] described a correlation between reduced SPARC expression and advanced clinical stage and poor outcome in primary BC. SPARC expression in metastatic tissue has not been reported. Both CXCL14 (CXC motive ligand 14) and TNFAIP3/A20 (tumor necrosis factor α induced protein 3) are involved in immunoregulatory and inflammatory processes. CXCL14 is a pro-migratory chemokine and plays an important role in tumor recognition by the immune system [13]. TNFAIP3 is a zinc-finger protein involved in the cytokine-mediated immune and inflammatory response mainly through the inhibition of nuclear factor NF-κB (NFκB) activation and tumor necrosis factor (TNF)-mediated apoptosis [25]. Down-regulation of CXCL14 and TNFAIP3 has been described in various primary tumor entities including breast tumors [13, 14, 26, 27], but to our knowledge, their role in metastasis has not been reported.

In conclusion, our study shows a down-regulation of *CADM1, RECK, SPARC, CXCL14* and *TNFAIP3* in brain metastases samples and especially implies CADM1 as an important prognostic factor, which is commonly lost in BC subtypes with poor outcome and refers to an increased risk of brain metastasis formation. The mechanisms leading to a loss of CADM1 expression are at least partially induced by promoter hypermethylation, a mechanism potentially reversible by therapeutic intervention. As loss of CADM1 seems to represent in general a more aggressive disease with a higher risk of relapse especially to the brain, these patients might need a more frequent follow-up including diagnostics such as regular clinical controls and early cMRI of the brain.

## MATERIALS AND METHODS

### Cell lines and patient material

For the methylation screening analysis, the human breast cancer cell line MDA-MB-231 (parental) and its bone- (SA) and brain-seeking (BR) variants were used. MDA-MB-231 was obtained from ATCC and passaged for a maximum of 6 months. MDA-MB-231(SA) cells were obtained from Prof. Theresa Guise and were comprehensively characterized by comparative genomic hybridization and genome-wide gene expression profiling as described in Pollari et al. (2011) [28]. The brain metastatic MDA-MB-231 variant was obtained from Prof. Toshiyuki Yoneda and was characterized in [29]. A non-tumorigenic epithelial cell line MCF 10A (ATCC, USA) was used as a control (see supplementary material). Authentications of all cell lines were conducted by short tandem repeat (STR) profiling to exclude cross-contamination between the cell lines.

For the qRT-PCR and methylation specific PCR (MSP) analyses, tumor tissue from 29 early stage primary BC patients and 21 BCBMs were collected from fresh frozen specimens (see supplementary material) [30]. Patient samples were obtained after surgical resection at the University Medical Center, Hamburg-Eppendorf (UKE), Germany. For the immunohistochemistry (IHC) analysis, three different sets of Tissue Micro Arrays (TMA) were used. The first large prognostic array (TMA I) consisted of 2197 primary breast cancer specimens operated in Basel, Switzerland [31]. Follow-up data were available for all patients with a median follow-up time of 61.0 months (range 7-171 months). The second TMA (TMA II) consisted of 243 invasive ductal carcinomas and 243 invasive lobular carcinomas operated in Tampere, Finland, with a maximum follow-up period of 19.8 years containing information on the site and time of tumor recurrence and overall survival [32]. A third TMA from UKE (TMA III) contained 89 cases with matched primary and lymph node metastasis samples [33]. In addition, whole section slides from 28 BCBM patients were also analyzed by IHC (see supplementary material). The REMARK criteria were followed for the patient and study set up, however no information about the treatment could be collected. All sample donors from UKE gave written informed consent to biological research into their samples as approved by the ethics committee of the chamber of physicians, Hamburg, Germany. The use of the Finnish tumor samples and patient records was approved by the Ethics Committee of the Pirkanmaa Hospital District. All clinical investigations have been conducted according to the principles expressed in the Declaration of Helsinki.

### 5-Aza-2'-deoxycytidine treatment of cells and isolation of total RNA and DNA

Different concentrations of 5-Aza-2'-deoxycytidine were tested for minimal toxicity (MTT assay) but full reversion of methylation. The concentration of 1 µM has been found as the most efficient one. Cells were cultured in absence or presence of 1 µM 5-Aza-CdR (n=3), and the medium was changed daily until the cells reached a confluence of 90-100%. Experiments were done in triplicates using different cell passages (see supplementary material).

Tumor tissue sections were manually dissected to obtain a tumor cell content of at least 70% [34]. Total RNA was isolated with the RNeasy Mini Kit (Qiagen, Hilden, Germany). DNA was extracted using InnuPREP DNA Microkit (Analytik Jena, Jena, Germany) (see supplementary material). The success of 5-Aza-2'-deoxycytidine treatment was verified by measuring the expression of methylated *MAGE-A1* and *RUNX3* genes [35, 36].

### Genome-wide expression analysis

The gene expression analyses of the cell lines and BCBM samples were carried out using the Whole Human Genome Oligo Microarray Kit, 4×44K (Agilent Technologies) (see supplementary material). The non-malignant cell line MCF 10A was used as a control for genotoxic stress response for the 5-Aza-2'-deoxycytidine treatment. The MIAME guidelines were followed in sample, array and data processing (see supplementary material). All array data are available at http://www.ncbi.nlm.nih.gov/geo GSE44354.

Potentially methylated genes in BC cells were defined using the following criteria: a) 2-fold expression difference between the treated and non-treated MDA-MB-231 BR cells, b) minimum expression value of 100 in one of the cell lines, and c) no expression change in the control cell line MCF 10A after the 5-Aza-CdR treatment.

The array data from nine BCBM samples were compared to 32 untreated primary breast tumors without relapse present in the GEO DataSet GSE21974 [37]. Differentially expressed genes were selected using the significance analysis of microarrays (SAM) algorithm with a false discovery rate of 5%. Genes with expression values above 100, which were at least 2-fold down-regulated in brain metastases as compared to primary tumors, were defined as potentially methylated.

The prognostic impact of *CADM1* expression was analyzed in two publicly available data sets (GSE3494 and GSE6532) including follow-up data for 201 and 217 patients respectively. The array data was median scaled whereas the *CADM1* expression was divided in quartiles and the two middle quartiles were combined for the survival analyses.

### cDNA synthesis and qRT- PCR

First Strand cDNA (Fermentas, St. Leon-Rot, Germany) was synthesized from 400 ng of total RNA. qRT-PCR reactions were run in duplicates and performed on the Mastercycler Eppendorf Realplex (Supplementary materials and Table 1). Data were analyzed by applying the ΔΔCT-method using RPLP expression for normalization. The results, expressed as fold changes, were set in relation to Universal Human Reference expression (see supplementary material).

### Methylation-specific PCR (MSP)

500 ng of genomic DNA were subjected to bisulfite treatment using the EZ DNA Methylation-Gold Kit (Zymo Research, Freiburg, Germany). DNA samples from bisulfite-treated MCF7 (positive) and HT29 (negative) cells were utilized as controls in the *CADM1* MSP. According to the methylation pattern, the results were categorized into wild type (WT), heterozygously methylated and homozygously methylated (see supplementary material).

### CADM1 IHC analysis

For CADM1 immunostaining, TMAs containing samples from primary tumors and lymph node metastases were employed. Additionally, 28 paraffin-embedded BCBM whole tissue sections were assessed. The rabbit polyclonal antibody anti-CADM1 (1:6000 dilution, S-4945, SIGMA-ALDRICH, Hamburg, Germany) was used and visualized using the DAKO ChemMate Detection Kit (#K 5001). The optimal dilution and pre-treatment was defined by testing well characterized positive (MCF7 and MDA-MB468) and negative (MDA-MB231, GI-101) formalin-fixed, paraffin-embedded (FFPE) cancer cell lines (for details see supplementary material). Normal tissue on the TMA with known negative (lymph nodes) and positive (normal epithelial cells of the colon and bronchus) protein expression of CADM1 served as positive and negative controls (see supplementary material).

Immunostaining was evaluated by two independent observers (HW and LW) and if discrepant findings were observed, the cases were reanalyzed together. The following parameters were taken into account: staining intensity (0-3), percentage of stained tumor cells (1-30% counted as 1, > 30-60% as 2, > 60-100% as 3) and the localization of staining in the tumor cells (membrane, nucleus). These scores were summed up to a total score, which was considered negative (score 0-1), weak (score 2-4) or positive (score 5-6).

### Statistical analysis

Comparisons in distribution of clinical and pathological variables were examined using the Chi-square-test (x^2^-test) or Fisher's exact test. P-values lower than 0.05 were considered statistically significant. Cumulative survival probabilities were assessed from the date of initial diagnosis until death or the date of the last follow-up and analyzed using the Kaplan-Meier-method and two-tailed log-rank test. Multivariate analysis was performed using the Cox proportional hazards model by including histology, hormone receptor status, HER2 status, age, tumor-size, pT, pN, pM and grade as confounding factors. All statistical analyses were performed using the SPSS software version 21 (Chicago, IL, USA).

## SUPPLEMENTARY MATERIALS AND METHODS FIGURE AND TABLES


