# Supplementary material for: Loss of CADM1 expression is associated with poor prognosis and brain metastasis in breast cancer patients
Source: Oncotarget. 2014 Mar 16;5(10):3076–87. doi: 10.18632/oncotarget.1832 (PMC4102793; doi:10.18632/oncotarget.1832)
Supplement: Supplementary file 1 [file oncotarget-05-3076-s001.docx]

**List of online supporting information:**

**Supplementary information:** Materials and methods.

**Supplementary Figure 1.** Expression of A) *CADM1*, B) *RECK*, C) *CXCL14*, D) *SPARC* and E) *TNFAIP3* (analyzed by qRT-PCR) in BCBM samples relative to primary breast tumors. P- values were determined by the log rank test. * p < 0.05, *** p < 0.01.

**Supplementary Table 1.** Primers (sequences given in 5`->3` direction) used in the qRT-PCR and MSP analyses as well as size of PCR product and annealing temperature.

**Supplementary Table 2.** Normalized gene expression values for 80 transcripts, which were found significant in both cell lines and patient samples.

**Supplementary Table 3.** Expression of *CADM1, RECK, CXCL14, SPARC* and *TNFAIP3* as analyzed by qRT-PCR in correlation to clinical parameters in primary BC and BCBM patients.

**Supplementary Table 4.** CADM1 membrane protein expression in correlation to clinical parameters in primary BC and BCBM patients in TMA II.

**Supplementary Table 5.** CADM1 nuclear protein expression in correlation to clinical parameters in primary BC and BCBM patients.

**SUPPLEMENTARY MATERIALS AND METHODS**

**Cell lines and cell culture**

The different MDA-MB-231 cell lines were grown in Dulbecco's Modified Eagle's Medium (DMEM) (PAA Laboratories, Pasching, Austria) supplemented with 10% inactivated calf serum, 1% penicillin/streptomycin, and nonessential amino acids at +37°C in a humidified incubator in an atmosphere of 5% CO2. MCF 10A cells were grown in DMEM/Ham's F-12 (PAA Laboratories) with 5% FBS, 10 μg/ml Insulin, 5 μg/ml Hydrocortisone, 20 ng/ml EGF, 100 ng/ml Cholera toxin and 2 mM L-Glutamine (all Sigma-Aldrich, Munich, Germany). The MCF10A cells were maintained in a humidified incubator at 37°C and 10% CO_2_. All cells were tested free of mycoplasma.

**Patient Material**

Patient samples were obtained after surgical resection at the University Medical Center, Hamburg-Eppendorf (UKE), Germany or operated in the University Hospital Tampere, Finland. The primary BCs used in the qRT-PCR validation analyses were of early stage of disease with a mean age of 62.2 years and none of them with distant metastasis at primary tumor resection. Five patients relapsed during the follow-up time (other sites than brain). In the BCBM patients the CNS relapse was diagnosed and operated at an average 75 months after the primary tumor operation. The large prognostic array (TMA I) consisted of 2197 primary breast cancer specimens with a median age of 62 (range 26–101) years. Survival data were either obtained from the cancer registry or collected from the patients attending physicians. The mean follow up time was 68 months (range 1–176). Tumor stage, lymph node status, grade, hormonal and HER2 status had a significant influence on survival (p < 0.001, data not shown). The patient samples were divided onto 4 slides each containing one sample per tumor measuring 0.6 mm in diameter. The second TMA (TMA II) consisted of 243 invasive ductal carcinomas and 243 invasive lobular carcinomas with a maximum follow-up period of 19.8 years, with detailed information of the site of relapse. The second TMA consisted of in total 6 slides containing one sample per tumor measuring 0.6 mm in diameter.

**5-Aza-2'-deoxycytidine treatment of cells and isolation of total RNA and DNA**

Cells were seeded at 10% (MDA-MB-231 and subtypes) or 20% (MCF 10A) confluence. 5-Aza-2'-deoxycytidine (5-Aza-CdR; Sigma-Aldrich St. Louis, MO) was dissolved in sterile water. Different concentrations of 5-Aza-2'-deoxycytidine (0.5, 1 and 5 µM) were tested for minimal toxicity (MTT assay) but full revision of methylation. A concentration of 1 µM was found as the most efficient one.

Total RNA from cell lines, primary BC and BCBM tissues was isolated with the RNeasy Mini Kit (Qiagen, Hilden, Germany) according to the manufacturer’s instructions. Manual dissection of tumor tissue section was used to obtain a tumor cell content of at least 70%. Sections were stained with 1% toluidin blue O (Sigma-Aldrich St. Louis, MO) and 0.2% methylene blue (Fluka, Germany). DNA was removed on the column with 7 U DNase I (Qiagen). For the quality control the integrity of the RNA was analyzed by agarose gel electrophoresis and/or Agilent 2100 Bioanalyzer (Agilent Technologies, Santa Clara, USA). DNA was extracted using InnuPREP DNA Microkit according to the manufacturer’s protocol (AnalytikJena, Jena, Germany). RNA and DNA concentrations were measured with the Nanodrop ND 1000 Spectrometer (PeqLab, Germany).

**cDNA synthesis and qRT- PCR**

The successful 5-Aza-2'-deoxycytidine treatment of the cells was proofed by testing the expression of methylated (*MAGE-A1* and *RUNX3*) and unmethylated (*GSTP1*) genes. 500 ng of cell line RNA was reverse transcribed using the SuperScript First Strand cDNA Kit (Invitrogen, Karlsruhe, Germany) according to the protocol. PCR was performed with a pre-incubation step at 95°C for 10 min, followed by 40 cycles of 95°C for 30 s, 58°C or 60°C for 45 s and 72°C for 45 s. Final elongation at 72°C was carried out for 7 min. *MAGE-A1, RUNX* and *GSTP1* PCR products were also separated and visualized by gel electrophoresis (Supplementary Table 2).

For five different genes a qRT-PCR was carried out to quantify the expression in primary breast cancer and BCBM samples. After total RNA extraction, first Strand cDNA (Fermentas, St. Leon-Rot, Germany) was synthesized following the manufacturer’s protocols from 400 ng of total RNA from both tumor entities and the universal human reference RNA (UHR). The mastermix consisted of 7.5 µl of SYBR Green (Fermentas, St. Leon-Rot, Germany), 0.4 µl (10^-5 µM) of each of the RT primer pairs (primer sequences in Supplementary Table 2) and 4.7 µl of RNase-free water. Each reaction mixture contained 13 µl of the mastermix and 2 µl of the template cDNA (1:30 dilution). Amplification was run in duplicates and performed using the Mastercycler Eppendorf Realplex with the following thermal cycling parameters: 10 min at 95°C, followed by 45 cycles of 95°C for 15 s, gene-specific annealing temperatures between 59 and 62°C for 30 s, and 72°C for 30 s. The transcripts of the RPLP0 gene were used as an endogenous RNA control. Data were analyzed by applying the ∆∆CT-method. The results, expressed as N-fold differences in target gene expression, were set in relation to UHR.

**Genome-wide expression analysis**

The gene expression analyses of the cell lines and BCBM samples were carried out using the Whole Human Genome Oligo Microarray Kit, 4x44K (Agilent Technologies). The non-malignant cell line MCF 10A was used as a control for genotoxic stress response for the 5-Aza-2'-deoxycytidine treatment.

500 ng RNA from untreated or 5-Aza-2'-deoxycytidine-treated cell lines of three independent experiments was pooled and labelled with Cy5-dCTPs (untreated) or Cy3-dCTPs (5-Aza-2'-deoxycytidine treated) with the Quick Amp Labeling Kit, two-color (Agilent Technologies) and hybridized on an array. The MIAME guidelines were followed in sample and data processing. Potentially methylated genes were defined based on the following criteria: a) 2-fold expression difference between the treated and non-treated MDA-MB-231 BR cells b) minimum expression value of 100 in the MDA-231 cell lines with 2-fold expression difference and c) no expression change in the control cell line MCF 10A after the 5-Aza-CdR treatment.

500 ng of tumor RNA from nine brain metastasis samples of breast cancer patients were similarly labeled and hybridized on the 4x44K Agilent microarray as the cell lines, according to the manufacturer’s instructions. The brain metastasis data were compared to array data from the 32 untreated primary breast tumors without relapse included in GEO DataSet GSE21974 (including 10 basal-like and 22 non-basal-like tumors) (Wikman et al., Bres Ca Res 2012). The arrays were quantile normalised and checked for systematic differences between the two array groups. Subsequently, differentially expressed genes were selected using the significance analysis of microarrays (SAM) algorithm with a false discovery rate of 5%. Potentially methylated genes were defined based on the following criteria: a) found significant in the cell line experiments described above, b) 2-fold downregulation in the brain metastases and c) minimum expression value of 100.

**Methylation-specific PCR (MSP)**

500 ng of genomic DNA from primary breast cancer patients, BCBM tissue samples and cell lines were subjected to bisulfite treatment using the EZ DNA Methylation-Gold Kit (Zymo Research, Freiburg, Germany) and eluted in 16 µl aliquots. Primers were designed to anneal specifically to the methylated or unmethylated bisulfite-modified DNA sequence within the target genes *CADM1* and *RECK* (Suppl. Table 2). Modified DNA was amplified in 25 µl reaction mixtures comprising 5 µl of PCR buffer, 0.5 µl respectively of 10 mM dNTP, forward and reverse primer, 16,87 µl of RNase-free water and 0,125 µl of EpiMark Hot Start Taq DNA Polymerase and 1,5 µl of BIS-DNA (New England BioLabs, Frankfurt am Main, Germany). MSP was carried out in a thermal cycler at 95°C for 30 s, followed by 40 cycles (denaturation at 95°C, annealing at gene and methylation specific temperatures, elongation at 68°C each for 20 s) and a final 5 min extension at 68°C. PCR products were separated in 2% agarose gels, stained with ethidium bromide and visualized under UV spectrophotometer. DNA samples from Bisulphite-treated MCF7 and HT29 cells were used as positive and negative controls in the *CADM1* MSP analyses. BT549 and T47D were used as positive and negative controls in the *RECK* MSP analyses, respectively. According to the methylation pattern, results were categorized into wild type (WT), heterozygously methylated and homozygously methylated.

**CADM1 IHC analysis**

For CADM1 immunostaining, different paraffin Tissue Micro Arrays containing a collective of 2683 primary breast carcinomas, 87 lymph node tissues and 20 non-malignant controls of different organs were employed. Additionally, 28 paraffin-embedded BCBM whole tissue sections were assessed. The first prognostic TMA samples were divided onto 4 slides each containing one sample per tumor measuring 0.6 mm in diameter. The second TMA consisted of in total 6 slides containing one sample per tumor measuring 0.6 mm in diameter., whereas the third smaller TMA with matched primary and lymph node metastases contained two samples each measuring 1.0 mm in diameter. The IHC was performed on 4um thick sections. The rabbit polyclonal antibody anti-CADM1, generated against the C-terminus of mouse SynCAM1 (Anti-SynCAM S-4945, SIGMA-ALDRICH, Hamburg, Germany), was used at a dilution of 1:6000 in DAKO ChemMate Antibody Diluent (DAKO ChemMate, Hamburg, Germany). The optimal dilution and pre-treatment was defined by testing well characterized positive (MCF7 and MDA-MB468) and negative (MDA-MB231, GI-101) formalin-fixed, paraffin-embedded (FFPE) cancer cell lines. After incubation at 60°C for 12 h, the sections were deparaffinised and rehydrated using xylene and serial dilutions of ethanol (100%, 96%, 80%), distilled water and 1xTBST buffer. Antigen retrieval was done in 1xBioGenex buffer in a steamer at 120°C for 5 min, followed by an overnight incubation at 4°C with the diluted primary antibody. The detection of protein expression was performed using the DAKO ChemMate Detection Kit (DAK). Expression was visualized using 3,3'-Diaminobenzidin (DAB, DAKO) as a chromogen and hematoxylin as a counterstain.

**Supplementary Figure 1.** Expression of A) *CADM1*, B) *RECK*, C) *CXCL14*, D) *SPARC* and E) *TNFAIP3* (analyzed by qRT-PCR) in BCBM samples relative to primary breast tumors. P values were determined by the log rank test. * p < 0.05, *** p < 0.01.

**Supplementary Table1:** Primer pairs for qRT-PCR and MSP analyses, sequences all given in 5`⇒3` direction

Wischnewski et al.,Mol Cancer Res. 2006 May;4(5):339-49.

Jiang et al., Pathobiology. 2008;75(4):244-51

Heller et al., Oncogene. 2006 Feb 9;25(6):959-68.

**Supplementary Table 2.** Normalized gene expression values for 80 transcripts, which were found significant in both cell lines and patient samples.

**Supplementary Table 3A.** *SPARC* qRT-PCR results and clinical association.

**Supplementary Table 3B.** *RECK* qRT-PCR results and clinical association.

**Supplementary Table 3C**. *CXCL14* qRT-PCR results and clinical association

**Supplementary Table 3D**. *CADM1* qRT-PCR results and clinical association

**Supplementary Table 3E**. *TNFAIP3* qRT-PCR results and clinical association

**Supplementary Table 4.** CADM1 membrane protein expression in correlation to clinical parameters in primary BC analyzed on TMAII. n.a. = not available, n.s. = not significant

**Supplementary Table 5.** CADM1 nuclear protein expression in correlation to clinical parameters in primary BC and BCBM patients.
